# Associations between psychological stress, discrimination, and oral health-related quality of life: the buffering effects of social support networks

**DOI:** 10.1590/0102-311XEN123123

**Published:** 2024-02-19

**Authors:** Luísa Santini Pinheiro, Irene Fanny Ritzel, Fernando Neves Hugo, Juliana Balbinot Hilgert, João Luiz Bastos, Roger Keller Celeste

**Affiliations:** 1 Universidade Federal do Rio Grande do Sul, Porto Alegre, Brasil.; 2 Simon Fraser University, Burnaby, Canada.

**Keywords:** Health-Related Quality of Life, Psychological Stress, Social Discrimination, Social Networking, Oral Health, Qualidade de Vida Relacionada à Saúde, Estresse Psicológico, Discriminação Social, Rede Social, Saúde Bucal, Calidade de Vida Relacionada con la Salud, Estrés Psicológico, Discriminación Social, Red Social, Salud Bucal

## Abstract

Stress and discrimination negatively affect quality of life, but social support may buffer their effects. This study aims: (1) to examine the associations between psychological stress, discrimination, and oral health-related quality of life (OHRQoL); and (2) to assess whether social support, stress and discrimination interact to modify their associations with OHRQoL. We used cross-sectional household-based data from a study including 396 individuals aged 14 years and over from families registered for government social benefits in a city in Southern Brazil. OHRQoL was measured with the *Oral Impacts on Daily Performance* (OIDP) scale; psychological stress was assessed with the *Perceived Stress Scale* (PSS); social support was assessed based on the number of close relatives or friends of the participant, and discrimination was assessed with a short version of the *Everyday Discrimination Scale*. Interactions were estimated using the relative excess of risk due to interaction (RERI). Adjusted effects were calculated with logistic regression. The prevalence of oral impacts among people with higher and lower PSS scores was 81.6% and 65.5%, respectively (p < 0.01). Social support was found to have no interactions with stress levels and discrimination. The association between social discrimination and OHRQoL (OIDP score > 0) was OR = 2.03 (95%CI: 1.23; 3.34) among people with a low level of stress, but was OR = 12.6 (95%CI: 1.31; 120.9) among those with higher levels (p = 0.09, for interaction). Individuals who reported experiencing higher levels of psychological stress and discrimination had worse OHRQoL; a synergistic effect with social support was not clear.

## Abstract (pt)

O estresse e a discriminação afetam negativamente a qualidade de vida, mas o apoio social pode amortecer seus efeitos. Os objetivos deste estudo são: (1) examinar as associações entre estresse psicológico, discriminação e qualidade de vida relacionada à saúde bucal (QVRSB); e (2) avaliar se apoio social, estresse e discriminação interagem para modificar suas associações com QVRSB. Os dados são de uma pesquisa transversal de base domiciliar de um estudo que incluiu 396 indivíduos com 14 anos ou mais de famílias registradas para benefícios federais em uma cidade no sul do Brasil. A QVRSB foi mensurada pelo *Impactos Orais no Desempenho Diário* (OIDP), enquanto o estresse psicológico foi avaliado pela *Escala de Estresse Percebido* (PSS). Além disso, o apoio social foi avaliado pelo número de parentes ou amigos próximos e a discriminação por meio da *Escala de Discriminação Diária* (EDS). As interações foram estimadas por meio do excesso relativo de risco devido à interação (RERI). Os efeitos ajustados foram calculados por meio de regressão logística. A prevalência de impactos bucais entre as pessoas com maior e menor escores de PSS foi de 81,6% e 65,5%, respectivamente (p < 0,01). O apoio social mostra interações inclusivas com níveis de estresse e discriminação. A associação entre discriminação social e QVRSB (escore OIDP > 0) foi OR = 2,03 (IC95%: 1,23; 3,34) dentre pessoas com baixo nível de estresse, mas foi de OR = 12,6 (IC95%: 1,31; 120,9) dentre aqueles com níveis mais altos (p = 0,09, para interação). Indivíduos que relataram níveis mais elevados de estresse psicológico e discriminação apresentaram pior QVRSB; o efeito sinérgico com o apoio social não foi evidente.

## Abstract (es)

El estrés y la discriminación afectan negativamente a la calidad de vida, pero el apoyo social puede mitigar sus efectos. Los objetivos de este estudio son: (1) examinar las asociaciones entre el estrés psicológico, la discriminación y la calidad de vida relacionada con la salud bucal (CVRSB); y (2) evaluar si el apoyo social, el estrés y la discriminación interactúan para modificar sus asociaciones con la CVRSB. Los datos provienen de una encuesta transversal de hogares cuyo estudio incluyó a 396 individuos de 14 años o más de familias registradas en beneficios del gobierno en una ciudad del Sur de Brasil. La CVRSB se midió mediante el *Impactos Orales en el Rendimiento Diario* (OIDP), mientras que el estrés psicológico se evaluó mediante la *Escala de Estrés Percibido* (PSS). El apoyo social se basó en el número de familiares o amigos cercanos, y la discriminación en la *Escala de Discriminación Cotidiana* (EDS). Las interacciones se estimaron mediante el excesivo de riesgo relativo debido a la interacción (RERI). Los efectos ajustados se calcularon mediante regresión logística. La prevalencia de impactos orales entre las personas con puntajes de PSS más altas y más bajas fue del 81,6% y del 65,5%, respectivamente (p < 0,01). El apoyo social presenta interacciones inclusivas con niveles de estrés y discriminación. La asociación entre discriminación social y OHRQoL (puntuación OIDP > 0) fue OR = 2,03 (IC95%: 1,23; 3,34) entre personas con un nivel bajo de estrés, pero fue OR = 12,6 (IC95%: 1,31; 120,9) entre aquellos con niveles más altos (p = 0,09, para interacción). Las personas que informaron tener niveles más elevados de estrés psicológico y discriminación tuvieron una peor CVRSB; el efecto sinérgico con el apoyo social no estaba claro.

## Introduction

Oral health is more than the absence of disease and necessarily includes patient-reported outcome measures such as oral-health related quality of life (OHRQoL) ^1^ ^,^ ^2^, as clinical measures are not sufficient to describe health. To assess the variability of OHRQoL, studies have evaluated the effects of stress ^3^, depressive symptoms ^4^ ^,^ ^5^ and support from social networks ^3^ ^,^ ^4^ ^,^ ^5^ ^,^ ^6^ ^,^ ^7^ ^,^ ^8^ ^,^ ^9^.

In 2010, USD 2.5 trillion was lost worldwide due to work-related disorders associated with psychological stress and related problems ^10^. Psychological stress occurs due to excessive demands related to the environment, life and work of the affected individuals ^11^. The person-context relation is dynamic, as the social environment is constantly changing, posing new demands and requiring continuous adaptability ^12^, which can result in chronic stress. This condition can impact OHRQoL ^3^ ^,^ ^9^ by causing individuals to engage in adverse health behaviors ^13^ ^,^ ^14^ ^,^ ^15^ ^,^ ^16^ such as alcohol consumption, smoking, and lower use of health services.

Discrimination is a highly stressful event that marginalized groups often face ^17^ ^,^ ^18^. People who are entitled to government benefits are particularly exposed to stigma and prejudice ^19^, experiencing stressors that affect their psychological and physiological health ^20^. A systematic review found an association between perceived discrimination and poor mental health, indicating that the impacts of discrimination on physical health are likely mediated by stress responses ^21^. Similarly, chronic stress has been identified as a mediator of the relationship between discrimination and mental health ^20^.

Social support networks may increase individuals’ abilities to deal with the negative effects of stress on health ^4^ ^,^ ^15^ ^,^ ^17^ ^,^ ^22^. There is an association between support from social networks and lower morbidity and mortality rates ^22^ ^,^ ^23^, better oral health ^3^ ^,^ ^12^ ^,^ ^24^, lower stress levels, and greater psychological well-being ^17^. Social support networks include the social relationships that each person maintains with others, such as intimate connections with family members and friends and formal relationships with external groups ^23^. They can have different implications, including the provision of social support among members ^25^ ^,^ ^26^. Social support includes qualitative dimensions of the social networks ^25^, which interact with other factors via psychological and behavioral mechanisms ^22^. Increased social support is associated with a reduced effect of discrimination on mental health among marginalized groups ^21^. Individuals with greater social networks are more likely to quit smoking and seek dental care more frequently ^27^ ^,^ ^28^. Material support from social support networks also seems to weaken the association between discriminatory experiences and alcohol abuse ^21^. Nonetheless, having close friends who consume alcohol, tobacco and marijuana is associated with greater chances of using these substances ^29^ ^,^ ^30^, as well as ultra-processed foods ^31^.

**Figure 1:**
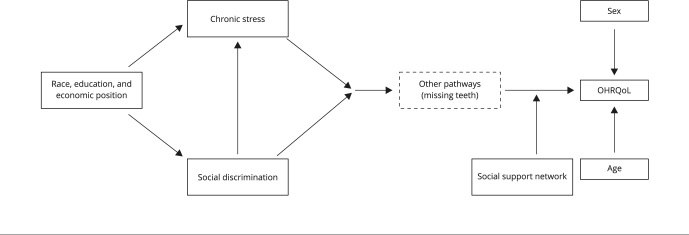
Analytical framework for the relationship of stress, discrimination, and social support networks with oral health-related quality of life (OHRQoL).

Based on the current understanding of these mechanisms, there might be an indirect relationship between social support and oral health, mediated by self-efficacy, that is, the belief an individual has in their capacity to deal with stressful situations ^32^. Social support networks may help people cope with stressful events and serve as a buffer against their negative impacts on oral health. A longitudinal study found that social support networks reduced stress levels and their impacts on OHRQoL ^3^. Limited support from social networks and high levels of psychological stress are associated with the adoption of unhealthy behaviors such as the consumption of alcohol and tobacco ^13^ ^,^ ^15^ ^,^ ^29^ ^,^ ^30^. Moreover, it has been postulated that when two or more factors (e.g., psychological stress and support from social networks) affect a health condition (e.g., OHRQoL) by the same mechanism (e.g., health behaviors and missing teeth), they can interact by antagonism or synergy ^33^ ^,^ ^34^: one factor can modify the effect of another (Figure 1). Although plausible, the hypothesis that social support networks interact with discrimination and stress by antagonism has not yet been well investigated; previous studies with adults and schoolchildren have shown that indicators of social capital reduced the effect of perceived stress on oral health ^35^ and that the sense of coherence reduced the effect of perceived discrimination on quality of life ^36^.

This study aims to: (1) examine the associations between psychological stress, discrimination, and oral health-related quality of life; and (2) assess whether social support, stress and discrimination interact to modify their associations with OHRQoL.

## Methods

This is a cross-sectional household-based study carried out in the city of São Leopoldo, Rio Grande do Sul State, Brazil. The *2010 Demographic Census* reported that the city had 64,561 resident families, and the target population consisted of the 17,922 families registered in the municipality’s Unified Register, which is used to obtain several social benefits. According to the register, 6,086 families received the Brazilian Income Transfer Program benefit and another 1,852 families were eligible for it but were not beneficiaries, totaling 7,938 families eligible for this study in 2016. Data were collected from November 2016 to August 2018; methodological information and quality control procedures were described in a previous study ^37^. The sample size was estimated to evaluate the difference in the prevalence of self-reported dental pain between the groups of people with and without the Brazilian Income Transfer Program benefit. Based on previous information, a sample of 767 individuals nested in 143 households was deemed necessary for each group, as everyone in the household would be included. Simple random selection was carried out using addresses provided by the Unified Register, and all data were collected in person by trained interviewers, using a standardized questionnaire administered at the respondents’ homes. Only respondents aged 14 or over were included in the analysis. The study was approved by the Research Ethics Committee of the Federal University of Rio Grande do Sul (UFRGS; protocol n. 1,269,053), in accordance with the principles of the *Declaration of Helsinki*.

### Outcome variable

OHRQoL was assessed with the *Oral Impacts on Daily Performance* (OIDP-9) scale, which contains nine items that measure the impact of oral conditions on daily activities, including physical, psychological, and social dimensions ^38^. The instrument has acceptable psychometric properties for use among adults ^39^ ^,^ ^40^ and adolescents ^41^. In the current analysis, the scale score was dichotomized into two results - OIDP = 0 and OIDP > 0 - in order to estimate the prevalence of the impacts, as suggested by Tsakos et al. ^42^.

### Main exposure variables

Three variables were studied as main exposures. Psychological stress was assessed using the *Perceived Stress Scale* (PSS-14) ^43^ ^,^ ^44^, which evaluates how individuals perceive stressful daily experiences. The PSS-14 has 14 questions with answers ranging from 0 to 4 (0 = never; 1 = almost never; 2 = sometimes; 3 = almost always; 4 = always). The final score ranges from 0 to 56, and higher values indicate higher stress. Final PSS scores were dichotomized into PSS < 39 and PSS ≥ 39 for analytical purposes.

Social support networks were evaluated based on the number of close relatives or friends of the participants, assessed using the following questions: “How many relatives do you feel comfortable with and can talk to about almost anything?” and “How many friends do you feel comfortable with and can talk to about almost anything?”; for which the possible answers were: none; one to two; three or more. For analytical purposes, the questions were combined, and participants were considered to have extensive social networks if they had three or more friends or relatives and to have limited social networks if they did not.

Discrimination was measured with a short version of the *Everyday Discrimination Scale* ^45^, which includes five dichotomous (yes, no) questions related to discriminatory experiences that may have occurred in the previous six months in/at: (1) the workplace; (2) home; (3) school/university; (4) interactions with police officers or security guards; and (5) public spaces. A follow-up question addressed the possible grounds for discrimination, including race/skin color, sex, religion, and socioeconomic status. In this study, any episode of discrimination reported in response to any question was considered as exposure to discrimination.

### Covariates

Five exogenous variables were included in the analysis as confounding factors (Figure 1). Sex (female, male), age (continuous variable later divided into three categories: 15-24, 25-44, ≥ 45, in years), race/skin color (dichotomized into two categories: white, non-white), education level (incomplete primary education, incomplete secondary education, complete secondary or tertiary education), and economic class, defined based on a set of household assets and divided into the following categories according to the Brazilian Economic Classification Criteria (Brazilian Association of Research Companies - ABEP): E (very low), D (low), C2 (lower middle) and C1 (middle).

The self-reported number of missing teeth was considered a mediator (Figure 1) and assessed with two questions, each referring to one jaw (upper or lower). Respondents were asked “Considering that a person has 16 teeth in the upper/lower jaw, how many teeth have you lost?”. The number of missing teeth in the upper and lower jaw were summed up, and the resulting variable was divided into three categories: 0-4, 5-27, and 28-32 missing teeth.

### Statistical analysis

The absolute and relative frequencies of OIDP > 0 were computed according to covariates and main exposures. Comparisons were presented and statistically tested with the chi-square test, incorporating the cluster effect, given that households were the primary sampling unit. Multiple logistic regression analysis was also performed to assess independent associations between covariates and exposure variables, stratified by two social network groups (extensive and limited). Odds ratios (OR) and 95% confidence intervals (95%CI) were estimated using two regression models. The results of the initial full model and a final model are presented in this article. The initial model included all interactions and the final model consisted of an adjusted analysis, including only the interactions and variables with p ≤ 0.10. The following interactions between variables of interest were estimated: (1) extensive social networks and high level of stress; (2) extensive social networks and discrimination; and (3) discrimination and high level of stress.

In addition to the interactions estimated by regression, the relative excess of risk due to interaction (RERI) was calculated as a departure from additivity of effects (prevalence scale). The RERI is a measure of interaction that reflects the proportional excess of risk/prevalence between the observed and expected proportions of a disease in a group exposed to both risk factors ^33^. All analyses were carried out using Stata software, version 16.1 (https://www.stata.com).

## Results

Over 410 addresses obtained from the Unified Register were contacted. Of these, 260 were valid, and 180 heads of household agreed to participate, which totaled 658 individuals. In total, 452 individuals aged 14 years and over were included in the analysis; however, due to missing data, the final analytical sample comprised 396 participants.

Table 1 shows that most participants in the sample were women (59.6%), aged from 25-44 years (42.2%), white (52.5%), and had complete secondary education (48.5%). While 12.4% of the participants reported experiencing high levels of psychological stress, 52.8% had limited social networks. The prevalence of oral impacts among women and men was 72.7% and 60%, respectively (p < 0.01). The prevalence of OIDP > 0 was 81.6% among participants who reported experiencing high levels of stress and 65.5% among those who reported experiencing lower levels of stress (p < 0.01). The prevalence of OIDP > 0 was 69.1% among people with limited social networks, but fell to 66.1% among those with extensive social networks (p = 0.53). Lastly, the prevalence of OIDP > 0 was 78.9% among those who reported experiencing discrimination and 61.1% among those who did not (p < 0.01).

**Table 1:** Absolute and relative distribution of *Oral Impacts on Daily Performance* (OIDP) according to covariates.

Parameter/Category	Total		OIDP = 0		OIDP > 0		p-value
	%	n	%	n	%	n	
Total	100.0	394	32.5	128	67.5	266	
Sex							< 0.01
Male	40.4	160	40.0	64	60.0	96	
Female	59.6	236	27.4	64	72.7	170	
Age (years)							0.24
15-24	27.5	109	38.5	42	61.5	67	
25-44	42.2	167	29.5	49	70.5	117	
≥ 45	30.3	120	31.1	37	68.9	82	
PSS (points)							< 0.01
< 39	87.6	347	34.5	119	65.5	226	
≥ 39	12.4	49	18.4	9	81.6	40	
Social support network							0.53
Limited	52.8	208	30.9	64	69.1	143	
Extensive	47.2	186	33.9	63	66.1	123	
Race/Skin color							0.98
White	52.5	201	32.5	65	67.5	135	
Non-white	47.5	182	32.6	59	67.4	122	
Discrimination							< 0.01
No	64.0	252	38.9	98	61.1	154	
Yes	36.0	142	21.1	30	78.9	112	
Brazilian Economic Classification Criteria							0.07
C1	18.1	75	40.9	27	59.1	39	
C2	42.2	175	35.8	58	64.2	104	
E-D	39.8	165	28.5	37	71.5	93	
Number of missing teeth							0.16
0-4	82.1	371	34.8	94	65.2	176	
5-27	14.2	64	27.7	28	72.3	73	
28-32	3.8	17	26.1	6	73.9	17	
Education level							0.22
Incomplete primary education	12.6	50	22.5	11	77.6	38	
Incomplete secondary education	38.9	154	33.8	52	66.2	102	
Complete secondary or tertiary education	48.5	192	34.0	65	66.0	126	

PSS: *Perceived Stress Scale*.

Tables 2 and 3 show stratified analyses by social support networks with the interactions. In the group with limited networks, there was a difference of 22.5 percentage points in the prevalence of oral impacts (OIDP > 0) between participants who reported experiencing low and high levels of stress (65.5% and 87.9%, respectively, p < 0.01, Tables 2 and 3). In contrast, a difference of 2.9 percentage points was observed between these groups when including only participants with extensive networks (65.9% and 68.8% for low and high levels of stress, respectively, p = 0.82). There was a non-statistically significant interaction by antagonism between higher levels of stress and extensive networks considering RERI = -0.30 (95%CI: -0.72; -0.12), which means that the observed prevalence was 30% lower than expected among the doubly exposed group.

**Table 2:** Prevalence of *Oral Impacts on Daily Performance* (OIDP) according to covariates stratified by social support networks.

Parameter/Category	Limited social network			Extensive social network		
	OIDP > 0 (%)	n	p-value	OIDP > 0 (%)	n	p-value
Total	69.1	208		66.1	186	
Sex			0.65			< 0.01
Male	67.1	73		54.7	86	
Female	70.2	134		76.0	100	
Age (years)			0.59			0.23
15-24	64.0	50		59.3	59	
25-44	69.2	94		73.2	71	
≥ 45	73.0	63		64.3	56	
PSS (points)			0.01			0.82
< 39	65.5	174		65.9	170	
≥ 39	87.9	33		68.8	16	
Race/Skin color			0.63			0.73
White	70.9	103		64.6	96	
Non-white	67.7	96		67.1	85	
Discrimination			0.06			< 0.01
No	64.3	129		58.2	122	
Yes	76.9	78		81.3	64	
Brazilian Economic Classification Criteria			0.57			0.37
C1	60.9	23		58.1	43	
C2	65.5	84		63.6	77	
E-D	71.3	80		72.0	50	
Number of missing teeth			0.73			0.33
0-4	67.6	139		63.1	130	
5-27	73.2	56		71.1	45	
28-32	66.7	12		81.8	11	
Education level			0.03			0.22
Incomplete primary education	84.4	32		64.7	17	
Incomplete secondary education	58.9	73		72.8	81	
Complete secondary or tertiary education	71.6	102		60.2	88	

PSS: *Perceived Stress Scale*.

**Table 3:** Prevalence (%) and prevalence ratio (PR) of impacts on *Oral Impacts on Daily Performance* (OIDP > 0) with the relative excess of risk due to interaction (RERI) between the size of social support networks, discrimination, and stress.

Parameter/Category	Limited social network			Extensive social network			RERI *
	%	n	PR (95%CI)	%	n	PR (95%CI)	
PSS (points)							
< 39	65.5	174	1.00	65.9	170	1.01 (0.86; 1.17)	-0.30 (-0.72; 0.12)
≥ 39	87.9	33	1.34 (1.14; 1.58)	68.8	16	1.05 (0.74; 1.48)	-
Discrimination							
No	64.3	129	1.00	58.2	122	0.91 (0.74; 1.10)	0.16 (-0.12; 0.43)
Yes	76.9	78	1.20 (1.01; 1.43)	81.3	64	1.26 (1.06; 1.51)	-
Parameter/Category	No discrimination			Discrimination for any reason			RERI *
	n	%	PR (95%CI)	n	%	PR (95%CI)	
PSS (points)							
< 39	60.4	227	1.00	75.4	118	1.25 (1.08; 1.45)	0.26 (-0.10; 0.61)
≥ 39	68.0	25	1.13 (0.84; 1.50)	95.8	24	1.59 (1.38; 1.81)	-

95%CI: 95% confidence interval; PSS: *Perceived Stress Scale*.

Note: RERI < 0 indicates antagonism, RERI > 0 indicates synergy.

* RERI: adjusted for age and sex.

Among individuals with limited social networks, 76.9% of those who experienced discrimination reported feeling some impact on daily performances, but only 64.3% of those who did not report experiencing discrimination felt this impact (p < 0.01) (Tables 2 and 3). In the subgroup with extensive networks, the prevalence of impacts (OIDP > 0) among those who reported and did not report experiencing discrimination was 81.3% and 58.2%, respectively (p < 0.01). Interaction indicators pointed to additive synergy: the prevalence of impacts in the doubly exposed group was 16% higher than expected, with a RERI = 0.16 (95%CI: -0.12; -0.43, after adjustment for age and sex, Table 3).

Table 3 shows the prevalence of OIDP > 0 according to stress and discrimination. In the group that reported a lower level of stress, 75.4% of those who experienced discrimination reported feeling some impact on daily performance, but only 60.4% of those who did not experience discrimination reported feeling this impact (p < 0.01) (Table 3). Among individuals experiencing high levels of stress, the prevalence of impacts (OIDP > 0) in those who reported and did not report discrimination was, respectively, 95.8% and 68% (p < 0.01). The interaction indicator pointed to additive synergy: the prevalence of impacts in the doubly exposed group was 26% higher than expected, with a RERI = 0.26 (95%CI: -0.10; -0.61, after adjustment for age and sex, Table 3).

Table 4 shows the results of the regression models. The initial full model included three joint interactions, and the final model only retained variables with p ≤ 0.10. The final model revealed that women were more likely to report an impact on OIDP (OR = 1.77, 95%CI: 1.13; 2.76). There were interaction terms between discrimination and stress, with p = 0.09. Among individuals with lower levels of stress, those who reported discrimination were found to have higher odds (OR = 2.03, 95%CI: 1.23; 3.34) of experiencing OIDP; among those with higher levels of stress, discrimination was associated with an OR = 12.6 (95%CI: 1.31; 120.9) for OIDP. Fit indicators showed that the final model with one interaction (Bayesian information criterion - BIC = 499.7) was better than the full initial model with three interactions (BIC = 521.5).

**Table 4:** Odds ratio (OR) and 95% confidence interval (95%CI) of *Oral Impacts on Daily Performance* (OIDP > 0) according to covariates in two regression models.

Parameter/Category	Initial full model			Final model		
	OR	95%CI	p-value	OR	95%CI	p-value
Social support network						
Limited	1.00					
Extensive	1.11	0.62; 1.99	0.72			
PSS (points)						
< 39	1.00			1.00		
≥ 39	1.93	0.56; 6.63	0.30	1.16	0.47; 2.85	0.74
Discrimination						
No	1.00			1.00		
Yes	1.61	0.78; 3.32	0.20	2.03	1.23; 3.34	0.01
Sex						
Male	1.00			1.00		
Female	1.51	0.93; 2.43	0.09	1.77	1.13; 2.76	0.01
Age (years)						
15-24	1.00					
25-44	1.43	0.80; 2.54	0.41			
≥ 45	1.07	0.50; 2.31				
Brazilian Economic Classification Criteria						
C1	1.00					
C2	1.21	0.65; 2.27	0.34			
E-D	1.64	0.82; 3.27				
Number of missing teeth						
0-4	1.00					
5-27	1.41	0.72; 2.75	0.52			
28-32	1.69	0.53; 5.40				
Education level						
Complete secondary or tertiary education	1.00					
Incomplete secondary education	0.78	0.46; 1.32	0.57			
Incomplete primary education	1.06	0.45; 2.51				
Interactions						
Social support network#PSS						
Larger#≥ 39 points	0.25	0.04; 1.53	0.13			
Social support network#D						
Larger#Yes	1.44	0.51; 4.09	0.49			
Discrimination#PSS						
Yes#≥ 39 points	6.33	0.62; 64.3	0.12	6.21	0.66; 58.1	0.09
Fit indices						
BIC	521.5			499.7		
Accuracy	67.2%			67.5%		
Hosmer-Lemeshow test	p = 0.59			p = 0.99		

PSS: *Perceived Stress Scale*.

## Discussion

Our findings indicate that there is an association between discrimination and high levels of psychological stress and OIDP. Additionally, we found a significant synergistic interaction between psychological stress and discrimination and OIDP. The presence of extensive social support networks resulted in inconclusive results that were not statistically significant; some results indicated that extensive social support networks interacted by antagonism with stress and by synergy with discrimination. The adjusted regression models did not support the proposed protective effect of extensive social networks on oral health related quality of life.

The lack of association between social support networks and OHRQoL in this sample differs from the current literature that reports that social support networks contributes to better oral health ^3^ ^,^ ^12^ ^,^ ^24^ and also differs from a study that showed that social capital can reduce the effect of psychological stress on oral health ^35^. People who have extensive social networks were also found to be more likely to report psychological well-being and positive OHRQoL ^15^. Despite assessing the size and composition of social support networks (friends and relatives), this study did not evaluate the qualitative aspects of these networks, such as the ability of the members to provide emotional or financial support. Our study includes a low-income population exposed to social vulnerability that generates major stressful events and reduces access to adaptation strategies. Therefore, our results may have been partly due to members of the social networks provided individuals with little instrumental/financial support. Social support networks also share health behaviors and mechanisms for adapting to stressful events, which should help decrease stress levels and improve OHRQoL. However, the coping strategies adopted by the participants are unknown and may not be beneficial in the long term. Based on the synergistic effect observed between the variables psychological stress and discrimination, the results confirm previous findings indicating an association between the variables ^17^ ^,^ ^20^ ^,^ ^46^. Discrimination causes stress and its main effects are related to psychological well-being ^20^ ^,^ ^21^ ^,^ ^46^. Previous studies have also found a higher prevalence of stress among Black individuals and indicated that stress mediates the relationship between discrimination and mental health ^20^ ^,^ ^47^. However, our study shows that the effects of stress and discrimination may be interdependent and that the effect of stress, in the absence of a discriminatory experience, may be insufficient to impact quality of life.

In the current sample, extensive social support networks also did not modify the association between discrimination and OIDP. Social participation may provide individuals with emotional support and decrease their stress levels, which might explain why it has been associated with better mental health indicators ^17^. Although emotional support seems relevant, economic support may be more important for maintaining good oral health, and it is scarce in a population that receives social benefits. Another possible explanation is that people who experience discrimination seek support from other sources.

One of the limitations of this study is that, as a cross-sectional analysis, we could not determine a temporal relationship between the factors. However, this does not exclude the possibility that worse oral disorders are a source of stressors and can lead to lower social participation ^48^. Furthermore, our findings show the importance of including the qualitative aspects of social support networks into analyses, in addition to their sizes. Another limitation is the lack of internal validity of the discrimination scale for this specific group ^49^. For example, there are concerns about the face validity of some items for populations that are out of school, only have temporary jobs, and work in public spaces or at home.

Generally, the results confirm previous findings on the positive association between higher levels of stress and worse OHRQoL, and do not confirm the hypothesis of interaction with social support networks. We also found an interaction between psychological stress and discrimination, which is a new and relevant aspect that can be better explored in future research. Our hypothesis is that individuals interact with the resources available in their context in different ways; therefore, the adaptation mechanisms they use to face daily challenges do not always generate health benefits and may not modify the impact of stress and discrimination on oral health-related quality of life. Future studies are needed to evaluate the relationship between OHRQoL and other social support indicators, focusing on the quantitative and qualitative aspects of social support networks.
